# Multilayer Cyclo-Olefin Polymer Films for Enhanced OLED Encapsulation

**DOI:** 10.3390/nano15201587

**Published:** 2025-10-17

**Authors:** Ji-Hoon Park, Kwan-Young Han

**Affiliations:** Department of Electronic and Electrical Engineering, Dankook University, 152, Jukjeon-ro, Suji-gu, Yongin-si 16890, Republic of Korea; parkzh902@naver.com

**Keywords:** cyclo-olefin polymer (COP), lifetime characteristics, nanolamination, OLED encapsulation, plasma treatment, water vapor transmission rate (WVTR)

## Abstract

The development of organic light-emitting diodes (OLEDs) for high-resolution, large-area displays relies on effective encapsulation technology. Accordingly, this study proposes a novel multilayer structure utilizing a cyclo-olefin polymer-based film. This solution significantly reduces process time and cost while achieving remarkable barrier performance. Optimization involved presenting various models and enhancing substrate–film adhesion via ultraviolet or plasma treatment, consequently improving water vapor transmission rate. Furthermore, the optimized structure’s feasibility as an OLED encapsulation layer was confirmed. These results promise to enhance core technological capabilities, improving production yield and minimizing costs—key factors for next-generation displays.

## 1. Introduction

Currently, organic light-emitting diodes (OLEDs) are garnering significant attention owing to their advantages, which include remarkable color purity, high contrast ratio, and low power consumption [1,2,3]. OLED devices utilize organic materials, enabling flexibility and allowing for thin, lightweight designs via their self-emissive principle. Leveraging these characteristics, displays in various form factors are becoming prevalent across various fields such as smartphones, tablets, and automobiles [4,5,6]. Among the technologies required to fabricate these OLED displays, the encapsulation process is crucial [7,8]. This encapsulation technology is the core technique for preventing moisture and oxygen from penetrating OLED devices and oxidizing their organic materials [9,10,11]. Conventional encapsulation technology uses a glass lid. Although this approach offers remarkable encapsulation performance, its thickness complicates flexible display applications [12,13]. Thin film encapsulation (TFE) technology was developed to address this limitation. TFE involves stacking thin films, typically ranging from hundreds of nanometers to several micrometers in thickness, to achieve lightweight, flexible structures with optimal barrier performance [14,15,16]. Because pinholes and microcracks can emerge when a single layer is deposited, organic and inorganic layers are alternately stacked to lengthen the penetration path for moisture and oxygen, thus enhancing barrier performance [17]. The inorganic layer blocks moisture and oxygen, while the organic layer prevents cracking, relieves stress, and provides flexibility [18,19]. Nevertheless, TFE technology has limitations: the alternating deposition of layers complicates manufacturing, increasing associated costs and time. Previously reported TFE technology adopted a nanolaminate structure by cross-stacking an inorganic layer (Al_2_O_3_, TiO_2_, and ZrO_2_) and an organic layer (Parylene-C and polyurethanes) [20]. This technology primarily uses ALD and CVD equipment and spin-coating equipment when employing solution processes. Changing the equipment for each encapsulation layer material increases the number of process steps, leading to higher time and cost requirements. Furthermore, achieving uniform deposition on large substrates and reliably implementing barrier performance remain challenging. Hence, various methods are being extensively researched to address these limitations [21,22,23]. A previous study fabricated a practical Al_2_O_3_ barrier using atmospheric pressure plasma-based spatial atomic layer deposition (ALD), enabling low-temperature and high-speed processing [21]. Another study proposed addressing these TFE limitations by utilizing Al_2_O_3_ and poly(ethylene glycol diacrylate) (pEGDA) thin films as the encapsulation layer [22]. The aforementioned study demonstrated adding hydrophobic functionality to the encapsulation surface to inhibit moisture penetration and verified large-area applicability by proving uniform deposition on substrates exceeding tens of cm^2^. In another study, process time and cost were minimized using a system that enables ALD and initial chemical vapor deposition (iCVD) within a single chamber [23]. By alternating deposition modes, the aforementioned study presented an organic/inorganic multilayer structure with remarkable barrier performance, optical transparency, and mechanical flexibility. Building upon these techniques, this study designed a multilayer polymer film based on cyclo-olefin polymer (COP) and employed it as the encapsulation layer in OLED devices to investigate its impact on lifetime characteristics in 60 °C/90% environments. Previously reported research results confirmed the potential of COP films as encapsulation layers by conducting reliability tests in 25 °C/50% environments [24]. In this study, to improve barrier performance, inorganic materials were deposited using ALD equipment, and polymer films with excellent WVTR performance, other than COP films, were adopted as encapsulation layers. Also, the two-stage encapsulation process reduces both costs and time, while the ability to freely control the composition of the polymer film layer offers significant potential.

## 2. Materials and Methods

### 2.1. Materials

Three polymer films were utilized to design the proposed multilayer structure based on COP. The core COP film, 25-µm thick, was provided by Zeon Korea (Seoul, Republic of Korea). A Polyethylene Naphthalate (PEN; ChemLab. Co., Ltd., Incheon, Republic of Korea) film, known for its excellent water vapor transmission rate (WVTR) characteristics, served as the sealing layer. Optical clear adhesive (OCA) (3M, Seoul, Republic of Korea) films were used to integrate PEN films, which lacked inherent adhesive power.

### 2.2. OLED Device Fabrication and Encapsulation Process

OLED devices were fabricated and encapsulated to assess the moisture and oxygen barrier properties of COP-based multilayer polymer films. First, a 1 × 1-inch indium tin oxide (ITO) glass substrate was cleaned in an ultrasonic cleaner with acetone, methanol, and isopropyl alcohol (IPA) for 5 min. Residual solution was discarded with a nitrogen blow gun, and the substrate was dried in a vacuum oven at 100 °C for 20 min to eliminate trace solvents. Subsequently, the substrate underwent 15 min of ultraviolet (UV)-ozone treatment to eliminate contaminants and enhance adhesion to the organic layers during vacuum thermal evaporation by activating -OH groups on the surface.

**Figure 1:**
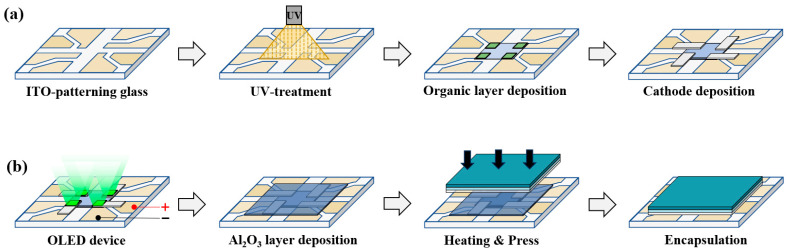
(**a**) Fabrication and (**b**) encapsulation processes of OLED devices.

Figure 1 illustrates the manufacturing and sealing processes of OLED elements. Green OLED devices are class/ITO/HAT-CN/CBP: Ir(ppy)_3_[10%]/TPBi/Alq_3_/LiF/Al structure. The manufacturing process proceeds with a series of steps. First, the cleaned ITO glass was transferred to a vacuum chamber, maintained at a base pressure of 5 × 10^−7^ Torr, to perform thermal vapor deposition. Hexaazatriphenylenehexacarbonitrile (HAT-CN) was deposited as the hole injection layer (HIL). Next, N,N′-Bis(1-naphthyl)-N′-bis(phenyl)-benzidine (NPB) was deposited as the hole transport layer (HTL). The light-emitting layer, utilized a host material, 4,4′-Bis(N-carbazolyl)-1,1′-biphenyl (CBP), simultaneously deposited with the emitter, Tris(2-phenylpyridine)iridium(III) (Ir(ppy)_3_). Then, 2,2′,2′ (1,3,5-Benzinetriyl)-tris(1-phenyl-1-H-benzimidazole) (TPBi) was deposited as the electron transport layer (ETL), and Tris(8-hydroxyquinolinato)aluminum(Alq_3_) as the electron injection layer (EIL).

After the organic layers were complete, the fine metal mask (FMM) was replaced. Subsequently, the lithium fluoride (LiF) and Al layers were deposited as buffer layers and cathodes. Following OLED element production, the sealing process proceeds, which utilizes a polymer film prepared beforehand. Regarding the COP and OCA films, the material’s inherent adhesive force ensures bonding after removing the protective film. To prevent an air layer from forming between the films, Roller Lamination equipment was employed. Before attaching the prepared polymer film to the OLED device, the OLED’s organic matter must be protected from the polymer film’s adhesive force. Consequently, Al_2_O_3_ thin films with barrier characteristics were deposited via ALD. In the ALD process, the N_2_ flow rate, controlled by the mass flow controller (MFC), was set to 50 sccm. To vaporize the Al_2_O_3_ thin film, trimethyluminum (TMA) was utilized as a precursor and H_2_O was employed as a reactant. Exposure time for TMA and H_2_O was 0.3 s, with a purge time of 20 s. Depositing a 50 nm thick film required approximately 6 h. The final sealing stage involved pressing the multilayer polymer film onto the Al_2_O_3_-coated OLED element inside a glove box under a N_2_ gas environment. Subsequently, thermal crimping was performed at 300 MPa for 150 s at 80 °C to complete the sealing process.

### 2.3. Measurements

Field emission scanning electron microscopy (FE-SEM, SIGMA500, Zeiss, Oberkochen, Germany) was utilized to analyze the emergence of dark spots after reliability tests. The mechanical properties of the polymer films were analyzed using a universal testing machine (UTM, AMETEK, Berwyn, PA, USA) to determine stress, strain, Young’s modulus, and other parameters. An oxygen/WVTR test system (C403H, Labthink Instruments Co., Ltd., Jinan, China) was employed to compare and analyze the WVTR characteristics of the polymer films. Surface comparison analysis between UV and plasma treatments was achieved utilizing an atomic force microscope (AFM; Jupiter XR, Oxford Instruments, Abingdon-on-Thames, UK). An I-V-L measurement system (Polaronix M6100, MacScience, Suwon, Republic of Korea) and a spectrometer (CS-1000, Minolta, Ramsey, NJ, USA) were employed to measure the OLED devices’ electrical and optical properties.

## 3. Results

**Figure 2:**
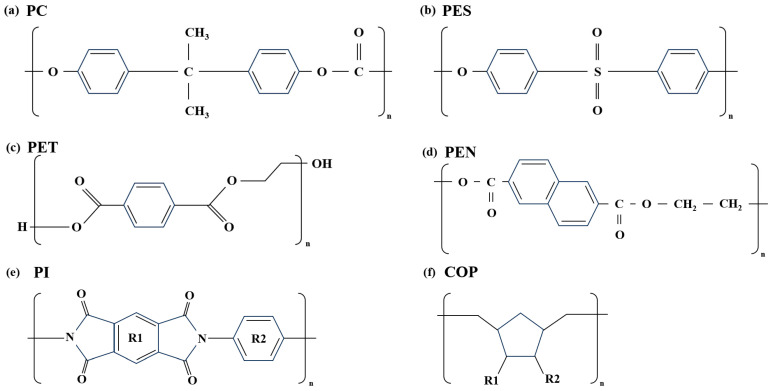
Chemical structure of polymer films: (**a**) PC, (**b**) PES, (**c**) PET, (**d**) PEN, (**e**) PI, (**f**) COP.

Figure 2 shows the chemical structures of polymer films. Figure 2a is a polycarbonate (PC) material, an amorphous aryl polymer containing carbonate bonds linking bisphenol A and carbonyl groups. Figure 2b shows a polyethersulfone (PES) material with a cross-linked structure of aromatic aryl (phenyl)-ether (-O-) and sulfonic (-SO_2_-) bonds. Due to their high T_g_ and excellent chemical resistance, they are being researched as candidates for flexible substrates in flexible displays [25]. Figure 2c shows a polyester film made from PET material, featuring ester bonds formed by the condensation of terephthalic acid and ethylene glycol. As a versatile material, it is used in a wide range of fields. Figure 2d shows PEN material, a polyester using naphthalene dicarboxylic acid instead of terephthalic acid. It has a higher T_g_ than PET and excellent moisture and oxygen barrier properties. Figure 2e shows a polyimide(PI) material exhibiting strong aromatic imide bonds formed by the condensation of aromatic diamines and aromatic dianhydrides. Among various polymer films, it has the highest T_g_ value and is widely used in the display field due to its excellent insulation properties and low dielectric constant. Finally, Figure 2f shows a COP material, a non-polar hydrocarbon-based amorphous polymer with a cyclic ring introduced into the main chain. It is gaining attention as a display material due to its excellent optical properties and barrier performance.

The polymer film selected as the barrier layer must be suitable for the OLED field. Accordingly, Table 1 summarizes the investigated characteristics of commercially available polymer films [26]. For OLED device applications, the glass transition temperature (T_g_) must exceed 100 °C, as the ALD process is performed at 80 °C inside a vacuum chamber. Therefore, the PET film is unsuitable as an encapsulation layer. The coefficient of thermal expansion (CTE) does not significantly impact the encapsulation process, as it is performed via thermal compression bonding after the multilayer polymer films are attached to the OLED device.

Regarding transmittance, although most films exhibit values in the 88–92% range, the PI film demonstrates low transmittance owing to its yellow coloration. Hence, the PI film is unsuitable for applications requiring high transmittance. When examining Young’s modulus values, most polymer films exhibit similar values. Finally, among the properties in Table 1, the most critical characteristics are oxygen transmission rate (OTR) and WVTR. PC and PES films are promising with properties like glass transition temperature and transmittance; however, their high OTR and WVTR values render them unsuitable as barrier layers. PEN and COP films exhibit the most favorable characteristics for oxygen and water vapor permeability. Hence, PEN and COP films were selected for the encapsulation layer role. Nevertheless, they do not satisfy the WVTR requirements for OLED devices when utilized as a single layer. Therefore, we attempt to verify their potential as an encapsulation layer by integrating them into a multilayer structure.

**Figure 3:**
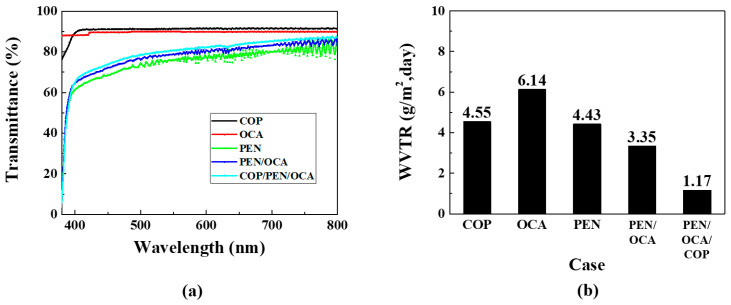
Mechanical property analysis of polymer films: (**a**) transmittance and (**b**) WVTR graphs.

First, it is necessary to verify the mechanical properties before applying the selected polymer films to the OLED device. Figure 3a presents the transmittance data for single layers of COP, PEN, and OCA films, including those for multilayer structures. The OCA film was utilized to attach the non-adhesive PEN film to the OLED device. The COP and OCA films exhibit high transmittance (approximately 91% and 90%, respectively), while the PEN film shows only approximately 80% transmittance in the visible light region. This characteristic also influences any multilayer structure containing the PEN film. The PEN film’s haziness is attributed to its molecular structure, which contains naphthalene rings [32,33,34]. These rings increase crystallinity, resulting in more significant differences in refractive index between crystalline grains and amorphous regions, which causes light scattering and reduced transparency. In addition, rapid cooling during film manufacturing can also cause refractive index inhomogeneity and light scattering. Although this haze phenomenon can be mitigated, as demonstrated by previous studies [35], we selected the PEN film as the barrier layer, prioritizing its oxygen and WVTR rates, while considering its transmittance performance.

Figure 3b presents the WVTR graphs for the polymer films. WVTR measurements were conducted according to the international ASTM F1249 standard [36]. For COP films, because existing commercial COP-based films exhibit differences in their material properties, verification via measurement equipment is necessary. Measurement results indicate that single-layer COP and PEN films exhibit similar WVTR characteristics at 4.55 and 4.43 g/m^2^·day, respectively. The OCA film exhibited the highest WVTR at 6.14 g/m^2^·day but was required for adhesion between both films and the substrate. The PEN/OCA multilayer structure demonstrated improved performance at 3.35 g/m^2^·day compared to the single-layer films. Finally, the PEN/OCA/COP multilayer film structure achieved the lowest WVTR at 1.17 g/m^2^·day. This reduction is partly attributable to the increased thickness from using three films, which enhances barrier performance; however, it also implies that the penetration path for moisture and oxygen is longer. Furthermore, the structure solely comprises polymer films, which ensures flexibility. Based on these results, various structures were designed and subjected to life tests to optimize the encapsulation layer.

**Figure 4:**
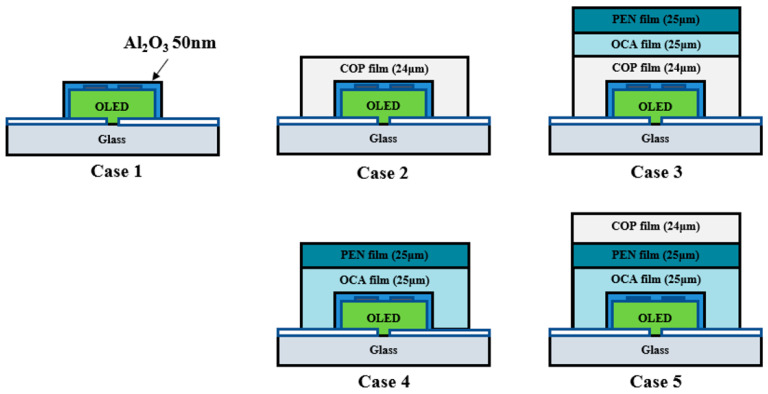
Schematic of the lifetime test for an OLED device employing a multilayer polymer film structure as an encapsulation layer.

Figure 4 presents various models for optimizing the multilayer polymer film’s barrier layer structure. The Al_2_O_3_ thin film used here functions as the inorganic layer, while the multilayer polymer film structure serves as the organic layer. Case 1 is a basic structure used to verify the barrier performance of a single layer of Al_2_O_3_ thin film (50 nm) deposited directly on the OLED device. This structure is expected to allow rapid penetration of moisture and oxygen due to inherent pinholes. Case 2 features a single layer of COP film deposited over the Al_2_O_3_ thin film, allowing evaluation of the extent of barrier performance improvement provided by the COP film compared to Case 1.

Case 3 models the structure with PEN/OCA attached over the COP film. Cases 2 and 3 enable a direct comparative analysis of lifetime test results based on the presence or absence of the PEN/OCA film. Case 4 features a structure with a PEN/OCA film attached directly over the Al_2_O_3_ thin film. This allows comparison of the barrier performance between the COP and PEN/OCA films when compared to Case 2.

Case 5 is designed with the most externally exposed COP film, enabling simultaneous comparison of Cases 3 and 4. Cases 3 and 5 elucidate the correlation between the polymer film and glass substrate interface, while Cases 4 and 5 enable a comparison of the blocking performance with and without the COP film. This design objective was applied to the OLED device structure, and a lifetime test was conducted at 60 °C and 90% humidity.

**Figure 5:**
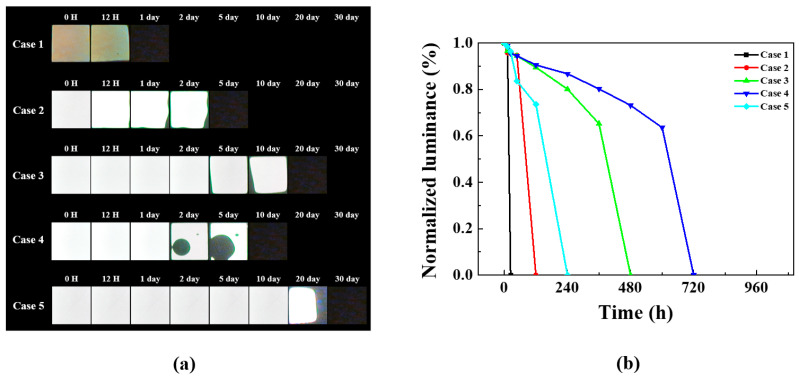
Comparative analysis of 60 °C/90% humidity life test results for Cases 1–5: (**a**) dark spot optical image and (**b**) normalized luminance graph over time.

Figure 5a presents the lifetime test results for the aforementioned cases. In Case 1, moisture and oxygen permeate through pinholes in the single-layer deposited Al_2_O_3_ thin film, resulting in degradation within a relatively short time. Case 2 features a structure in which a COP film is encapsulated directly over the Al_2_O_3_ thin film. The COP film, functioning as an organic layer, blocks pinholes in the inorganic layer, thus improving barrier properties. Furthermore, unlike Case 2, which utilizes a COP film, Case 4 exhibits an emissive area persisting up to 5 days, but dark spots due to pinholes emerge after Day 2. Cases 3 and 5, comprising multilayer polymer films, exhibit better barrier properties than other cases. This effect is ascertained to be similar to increasing moisture and oxygen penetration pathways by cross-stacking organic/inorganic layers.

Figure 5b shows the luminance changes of all devices over time in a graph. Figure 5b reveals a sudden performance degradation in all devices. Dark spots appeared on all devices before complete degradation occurred. The observation of dark spots indicates the step where moisture and oxygen permeate and contact the organic layer. The organic layer, upon contact with moisture and oxygen, rapidly degrades, causing dark spots and ultimately leading to degradation. Furthermore, the reason each device exhibits a different rate of performance degradation is due to the differing encapsulation layer structures applied to each device. The encapsulation layer applied in Case 1 was a single-layer Al_2_O_3_ thin film without an additional barrier layer, leading to complete degradation after 12 h. The encapsulation layers applied in Cases 2–5 featured a multilayer structure of inorganic and polymer films, capable of suppressing diffusion even if moisture and oxygen permeate. This explains why the rate of performance degradation differed across each device.

When comparing Cases 3 and 5, the structure with the COP film is positioned at the top of the encapsulation layer and exhibits a longer lifetime than the structure positioned at the bottom. This difference is also evident in Figure 5b, where Case 3 reached its half-life in approximately 400 h, while Case 5 exceeded 600 h. This disparity suggests materials are affected differently by the 60 °C, 90% relative humidity environment, necessitating root cause analysis. Although Case 5 has the best barrier properties, it still fails to satisfy the WVTR performance required for OLED applications. Consequently, root cause analysis was conducted.

**Figure 6:**
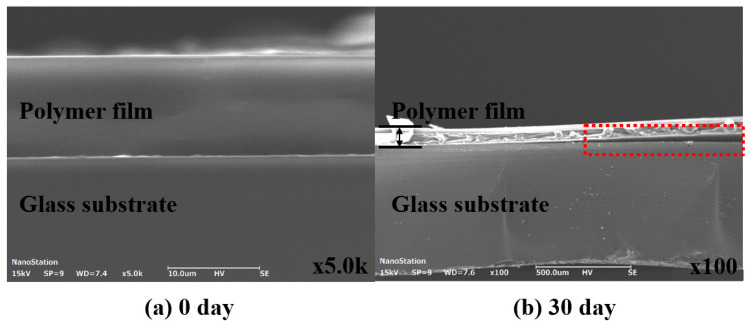
Cross-sectional image comparison analysis of life test samples: (**a**) 0 day and (**b**) 30 day.

Figure 6 presents SEM images of the cross-section before and after 30 days of Case 5’s life test. Figure 6a illustrates the pre-test cross-section, confirming that the interface between the polymer film and the glass substrate is fully adhered, which indicates no deformation during the heat pressing encapsulation process. Figure 6b shows the cross-section after the 30-day life test (at 60 °C and 90% humidity), revealing delamination between the polymer film and the glass substrate. This delamination is attributed to the permeation of moisture and oxygen over time, which reduced the adhesive strength. Consequently, the adhesive strength was further verified over the life test duration to highlight the cause.

**Figure 7:**
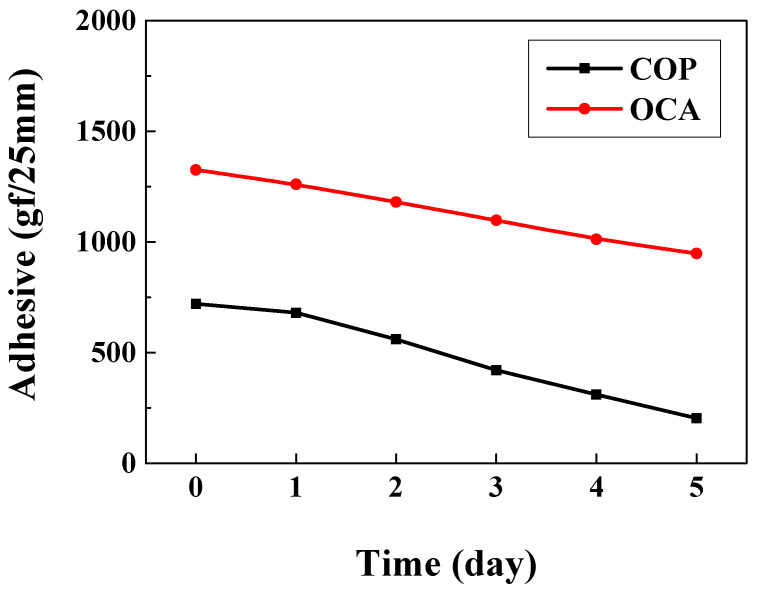
Adhesion strength comparison graph over lifespan test time.

To compare adhesion strength over time during the lifespan test, adhesion was measured according to the ASTM D3330 standard [37]. A 90° peel test was performed after attaching COP and OCA films to a steel use stainless (SUS) substrate. Because simple roll lamination yielded a significantly low adhesion force (101.9 gf/25 mm) for the COP film, a thermal compression process (80 °C, 300 MPa) was conducted instead. Similarly, the OCA film was attached via thermal compression to simulate its application as an encapsulation layer on an OLED device. The samples were placed in a constant temperature and humidity chamber (60 °C, 90% humidity) to analyze alterations in adhesion strength over the life test period. OCA’s initial adhesion (1325.6 gf/25 mm) decreased to 948 gf/25 mm over time. COP’s initial adhesion (720.4 gf/25 mm) decreased significantly to 203.9 gf/25 mm after 5 days (Figure 7). These results suggest that the film’s adhesive components undergo hydrolysis or weakening under harsh conditions [38]. Hence, enhancing adhesion is necessary to improve OLED device lifespan. Among various approaches, we analyze the effects of UV or plasma treatment as potential improvement strategies.

**Figure 8:**
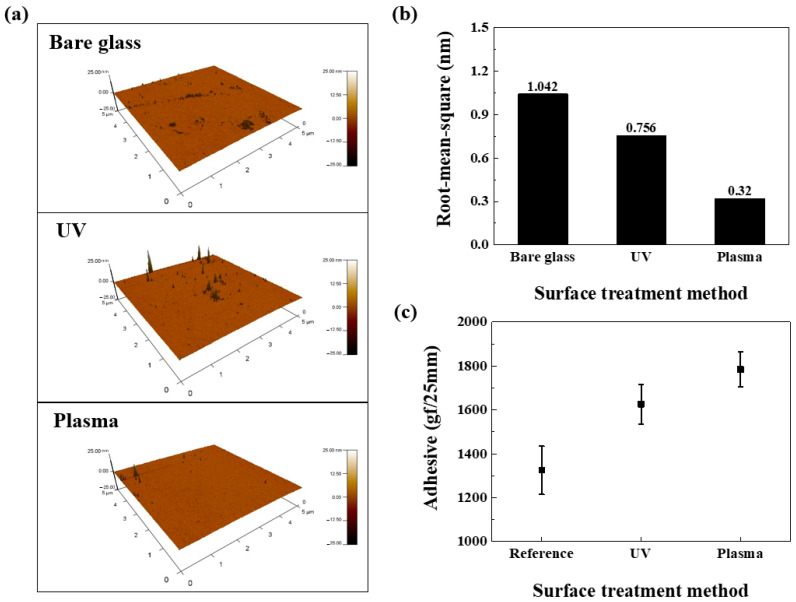
Comparison of RMS roughness via STM: (**a**) AFM scanning image, (**b**) RMS roughness comparison graph, and (**c**) adhesion strength comparison graph by STM.

Figure 8 illustrates data measuring surface roughness alterations after UV or plasma treatment is applied to a glass substrate. Figure 8a presents 3D scanning images measured utilizing an AFM instrument, comparing bare (untreated), UV-treated, and plasma-treated glass substrates. Based on these images, Figure 8b illustrates the root-mean-square (RMS) roughness alterations by the surface treatment method (STM). The UV-treated substrate’s RMS is 0.756 nm (a 37.8% improvement over bare glass). The plasma-treated substrate’s RMS is 0.32 nm (a 69.3% improvement). When surface modification is performed, there is a tendency for the RMS to generally decrease with surface modification, with plasma treatment exhibiting the highest improvement rate. The decrease in RMS is attributed to the breaking of chemical bonds in organic molecules, thereby discarding impurities [39,40,41]. Plasma treatment can modify the glass substrate surface without damage, making it suitable for use on the ITO glass substrate of OLED devices. However, to prevent damage to the OLED’s organic layer, a mask is utilized during treatment, ensuring surface modification occurs only where the polymer film is attached. Additional analysis was conducted on the surfaces of these treated substrates.

Figure 8c shows that the reference sample (25-μm-thick OCA film on untreated SUS substrate) exhibited an adhesion force of approximately 1325 gf following the 180° peel test. UV- and plasma-treated samples, where the SUS substrate received treatment before OCA attachment, exhibited adhesion forces of 1626 and 1784 gf, respectively. Compared to the reference sample, adhesion strength gradually improved, with the plasma-treated sample exhibiting the highest adhesion strength. Regarding the UV treatment case, this improvement stems from surface treatment creating hydrophilic groups (-OH, -CHO, and -COOH) [42]. The resulting high surface energy increases reactivity, enhancing the bond between the film and substrate, thus improving adhesion. In plasma treatment, high-energy species (electrons, ions, and radicals) react to coat the substrate with polar functional groups. The polar functional groups render the substrate surface hydrophilic, increasing its reactivity and consequently improving adhesion [43]. We propose applying this mechanism to the OLED encapsulation layer to enhance barrier performance.

**Figure 9:**
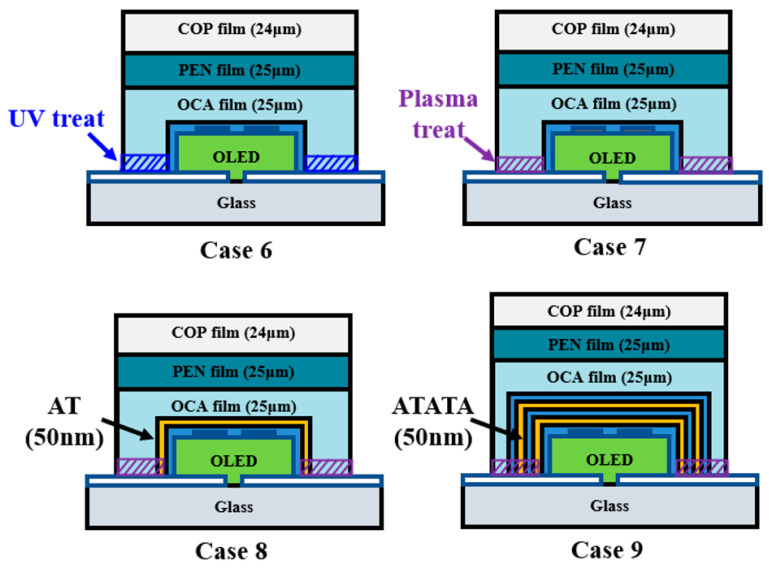
Schematic of improved COP-based multilayer high-barrier film encapsulation design.

Figure 9 illustrates a novel model proposed to address delamination. Case 6 enhanced adhesion between the substrate and film by increasing the substrate’s surface energy via UV treatment. Although UV treatment is usually part of ITO cleaning, its effect is transient. Therefore, after fabricating the OLED device, an Al_2_O_3_ thin film was deposited, followed by masking to protect the organic materials from UV. This increased the substrate’s surface energy, enhancing film adhesion. Case 7 involved plasma treatment, producing OLED devices via a process similar to UV treatment. Case 8 addressed the pinholes observed in Case 4 by depositing a 25 nm Al_2_O_3_ thin film followed by a 25 nm TiO_2_ thin film on top of the encapsulation layer. Case 9 involved cross-stacking Al_2_O_3_ and TiO_2_ films (10 nm thickness each, 50 nm total thickness) with identical processing time. This nanolamination mechanism for the Al_2_O_3_ and TiO_2_ films prevents pinholes by stacking different inorganic materials, as each layer inherently possesses pinholes [44]. A lifespan test was conducted on this improved encapsulation layer structure at 60 °C and 90% relative humidity.

**Figure 10:**
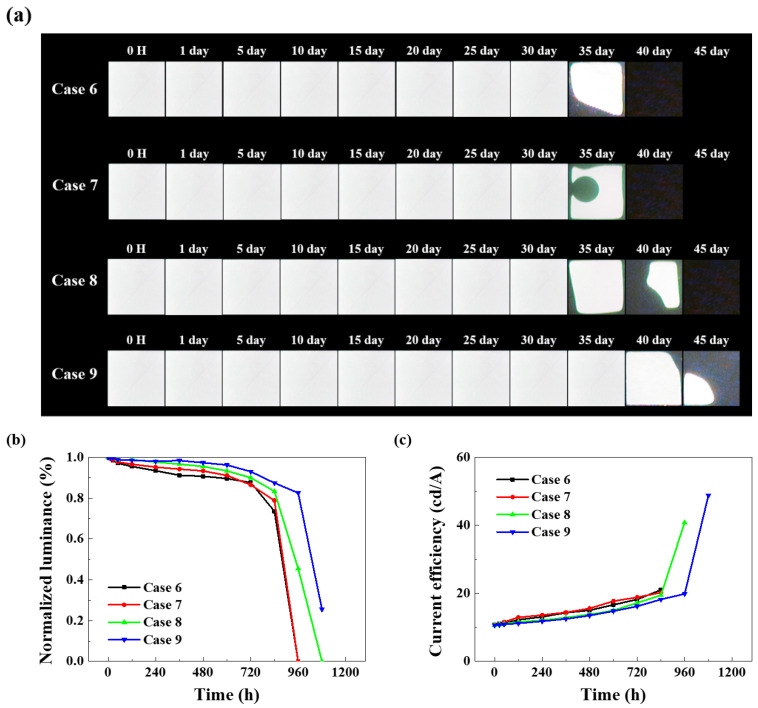
Comparison of electrical and optical characteristics of OLED devices with improved encapsulation structure: (**a**) dark spot optical image, (**b**) normalized luminance graph over time, (**c**) current efficiency–time characteristics.

Figure 10a presents the lifetime test data for OLED devices using an improved COP-based multilayer polymer film structure. UV and plasma treatments were applied to enhance adhesion between the substrate and surface. Hence, unlike the previous Cases 1–5, operation without dark spots was observed for up to 30 days. Figure 10b confirms that Cases 6–9 exhibit a delayed time to reach half-life, demonstrating enhanced lifetime characteristics with the overall improved encapsulation layer. Comparing Cases 6 and Case 7, the UV and plasma treatments exhibit no significant difference in lifetime test results. This indicates that while improved adhesion affects WVTR, the numerical differences between STMs are negligible. However, plasma treatment is preferred due to advantages like shorter processing time and longer-lasting surface modification effects than UV treatment, rendering it a viable option for continued use. Case 8 is designed to prevent pinholes and enhance moisture and oxygen barrier properties by cross-stacking Al_2_O_3_ and TiO_2_ thin films. During Case 8’s life test, a tendency for dark spots to expand was observed from Day 35. Unlike Cases 4 and 7, no circular dark spots formed at the luminescent area’s center. This suggests that the structure, in which Al_2_O_3_ and TiO_2_ films are each laminated at 25 nm thickness, increases the penetration pathways for moisture and oxygen.

Figure 10b enables a comparative evaluation of the normalized luminance of each device after reliability testing in a 60 °C/90% humidity environment. All devices were operated at a voltage of 9 V, with an initial luminance of 2000 (±100) cd/m^2^. Case 9, featuring the Al_2_O_3_/TiO_2_ nanolaminate structure, exhibits a half-life of approximately 1000 h.

Figure 10c shows the current efficiency graph over time during reliability testing. Generally, the current efficiency of all devices exhibits an upward trend, as the rate of current decrease is greater than the rate of luminance decrease. The reason for the greater current decay rate is multifaceted, but over time, dark spots and locally shorted areas within the light-emitting region can cause the overall current to decrease [45,46]. The initial current efficiency of all devices was similar at 10.7 cd/A. At 1080 h, Case 9 exhibited the highest measured current efficiency at 48.8 cd/A.

Based on these results, we were able to compare and evaluate the lifetime characteristics and electrical properties according to the encapsulation structure. Especially, this nanolaminate structure significantly enhances lifetime performance by compensating for pinholes while blocking moisture and oxygen. Previously reported research used a nanolaminate structure composed of an inorganic layer (Al_2_O_3_) and an organic layer (alucone and parylene C) as the encapsulation layer. Although reliability test conditions varied, the time to reach the half-life was mostly 190 h or 380 h [11,20]. Comparing these results, the time to reach half-life for Case 9 proposed in this paper is approximately 1000 h, showing a difference of about 3 to 5 times. 

## 4. Conclusions

This study utilized a COP-based multilayer polymer film structure to shorten existing process steps while improving barrier performance. To optimize the multilayer polymer film structure, several encapsulation models were proposed and applied to OLED devices for reliability testing. The results obtained demonstrated that the Glass/OCA/PEN/COP structure exhibited no dark spot formation for up to 10 days (under 60 °C, 90% humidity); however, its WVTR was insufficient for OLED device application, prompting root cause analysis. The root cause analysis identified two issues: reduced adhesion between the substrate and film during the lifetime test resulted in delamination and subsequent moisture and oxygen ingress. In addition, pinholes in the single-layer Al_2_O_3_ film caused central dark spots. To address these limitations, plasma treatment was adopted to enhance adhesion. Barrier performance was further improved by employing a nanolaminate structure combining Al_2_O_3_ and TiO_2_ films. Consequently, the plasma-treated Glass/OLED/Nanolaminate layer/OCA/PEN/COP device reached its half-life at approximately 1000 h and demonstrated the best barrier performance, sustaining no dark spot formation for up to 35 days.

Although material characteristics impose certain limitations, leveraging these results for future improvements is expected to significantly minimize the time and cost of OLED device fabrication, substantially impacting its economic viability.

## Figures and Tables

**Table 1:** Comparative analysis of numerical values for mechanical properties of polymer films.

Materials	T_g_ (°C)	CTE (ppm/°C)	Transmittance (%)	O_2_ Permeability (cc, 100 µm/m^2^, Day)	H_2_O Permeability (g, 100 µm/m^2^, Day)	Young’s Modulus (GPa)	Ref.
Glass	690	8	91	-	-	73	-
PC	155	70	90	900	50	2.1–2.4	[27]
PES	223	60	89	650	105	2.4–8.6	[28]
PET	78	30	89	18	9	2.5–3.0	[29]
PEN	121	20	88	1.0–3.0	5.77	6.1	[30]
PI	340	50	30	-	135	2.8	[31]
COP	138	60	92	0.01–0.1	0.1–0.5	2.7	[31]

## Data Availability

The data is contained within the article. For further reasonable inquiries, please contact the corresponding author.

## Abbreviations

The following abbreviations are used in this manuscript:

OLEDs	Organic light-emitting diodes
COP	Cyclo-olefin polymer
WVTR	Water vapor transmission rate
OTR	Oxygen transmission rate
TFE	Thin film encapsulation
ALD	Atomic layer deposition
OCA	Optical clear adhesive
STM	Surface treatment method
SUS	Steel use stainless
AT	Al_2_O_3_/TiO_2_
ATATA	Al_2_O_3_/TiO_2_/Al_2_O_3_/TiO_2_/Al_2_O_3_
